# Hospital infections and health-related quality of life after cardiac surgery: a multicenter survey

**DOI:** 10.1186/s13019-024-02559-4

**Published:** 2024-02-10

**Authors:** Hilda G. Rijnhart-de Jong, Jo Haenen, Fabiano Porta, Marijke Timmermans, E. Christiaan Boerma, Kim de Jong, on behalf of the participating centers of the Cardiothoracic Surgery Registration Committee of the Netherlands Heart Registration, S. Bramer, E. J. Daeter, G. J. F. Hoohenkerk, A. L. P. Markou, R. G. H. Speekenbrink, P. Segers, W. Stooker, W. W. L. Li, J. A. Bekkers, F. Porta

**Affiliations:** 1 Department of Cardiothoracic Surgery, Medisch Centrum Leeuwarden, Henri Dunantweg 2, Leeuwarden, 8934 AD The Netherlands; 2 Department of Intensive Care, Leeuwarden Medical Centre, Leeuwarden, The Netherlands; 3 Department of Epidemiology, Leeuwarden Medical Centre, Leeuwarden, The Netherlands; 4 Netherlands Heart Registration (NHR), Utrecht, The Netherlands; 5 Department of Sustainable Health, Rijksuniversiteit Groningen, Campus Fryslân Leeuwarden, Leeuwarden, The Netherlands

**Keywords:** Health-related quality of life, Cardiac surgery, Multicenter registry, Hospital infections, Physical recovery

## Abstract

**Background:**

Recent research suggested that hospital infections are a predictive marker for physical non-recovery one year after cardiothoracic surgery. The purpose of this study was to explore whether this risk factor is etiologic. Additional, the influence of a potential effect modifying factor, diabetes mellitus, was investigated.

**Methods:**

In this multicenter study, patients underwent elective or urgent cardiothoracic surgery between 01-01-2015 and 31-12-2019, and completed pre- and one year post-operative Short Form Health Survey 36/12 quality of life questionnaires. A binary logistic regression model, in which the inverse of the propensity score for infection risk was included as a weight variable, was used. Second, this analysis was stratified for diabetes mellitus status.

**Results:**

8577 patients were included. After weighing for the propensity score, the standardized mean differences of all variables decreased and indicated sufficient balance between the infection and non-infection groups. Hospital infections were found to be a risk factor for non-recovery after cardiothoracic surgery in the original and imputed dataset before weighting. However, after propensity score weighing, hospital infections did not remain significantly associated with recovery (OR for recovery = 0.79; 95% CI [0.60–1.03]; *p* = 0.077). No significant interaction between diabetes mellitus and hospital infections on recovery was found (*p* = 0.845).

**Conclusions:**

This study could not convincingly establish hospital infections as an etiologic risk factor for non-improvement of physical recovery in patients who underwent cardiothoracic surgery. In addition, there was no differential effect of hospital infections on non-improvement of physical recovery for patients with and without diabetes mellitus.

*Trial registration* International Clinical Trials Registry Platform ID NL9818; date of registration, 22-10-2021 (https://trialsearch.who.int/).

**Supplementary Information:**

The online version contains supplementary material available at 10.1186/s13019-024-02559-4.

## Background

Health-related quality of life (HRQoL) before and after surgery is recognized as important outcome [1–4], and general improvement in health related quality of life (HRQoL) in the overall cardiac surgery population is well-documented [5, 6]. At the same time, a substantial percentage of patients does not perceive an improvement in HRQoL after cardiac surgery [4, 7]. These seemingly contradictory findings fuel the search for risk factors that characterize subgroups of patients that do and do not report improvement in HRQoL after cardiac surgery. Various studies identified predictive factors for physical non-recovery in this setting, including female sex, diabetes mellitus (DM) and age [8, 9]. Although such findings are of interest, many of these studies are not aimed at identifying modifiable etiologic risk factors, and therefor lack the potential for a meaningful intervention [10]. Recently, our group identified hospital infections as a predictive marker for physical non-recovery after cardiac surgery [11]. Due to the single center explorative character of the study further confirmation felt obligatory to guide future interventions. Besides, the mentioned study did not consider confounding factors. To this end we performed a multicenter survey in the Netherlands that aimed to explore whether peri- and postoperative hospital infections during hospital stay are an etiologic risk factor for non-improvement of physical HRQoL after cardiac surgery. Since it is clear that DM makes patients vulnerable to hospital infections and may additionally change its course, the influence of this potential effect modifying factor was additionally investigated [12].

## Methods

### Setting and study population

In this retrospective study, all adult patients who underwent elective or urgent (i.e. those who require an intervention for medical reasons within the current intake) cardiothoracic surgery between 01-01-2015 and 31-12-2019 and completed pre- and one year post-operative Short Form Health Survey 36-version 2 or Short Form Health Survey 12 quality of life questionnaires (SF 36-2/SF 12) were included. Data were extracted from the Netherlands Heart Registration (NHR), a nationwide registry of all invasive cardiac interventions, comprising data from all Dutch hospitals [13]. The NHR facilitates value-based outcome monitoring, including quality of life outcome. Participating hospitals are responsible for data collection and registration and check their own data. The NHR analyses patient data, provides online dashboards and reports relevant outcome indicators in yearly, publicly accessible reports. Each year, within the NHR, data validation and verification is performed by standardized quality controls and monitoring visits (audits). In addition, the distribution of patient-relevant outcomes between hospitals is observed to verify that no striking differences exist. In the case of a significant variation in outcomes, processes of healthcare delivery are discussed and good practices are shared [14]. Datasets consist of a mandatory standard part (with a 90% completeness requirement) and a voluntary part, including HRQoL [13–15]. Within this study, mainly data of the mandatory standard set was used. This study complies with the Declaration of Helsinki and Good Clinical Practice guidelines. The study protocol is available at the International Clinical Trials Registry Platform, under main ID NL9818 [16]. The study was approved by the institutional review board MEC-U (W19.270) and conducted in agreement with the principles of the Declaration of Helsinki. A waiver for informed consent for analysis with the data of the NHR data registry was obtained. Preoperative, intraoperative- and postoperative data with a one-year follow-up period were collected and stored in a pseudonymized database.

### Measurement of the HRQoL and definitions

The SF-36-2 is a standardized, validated and widely used HRQoL assessment tool [17]. The SF-12 is a validated shortened version of the SF-36 questionnaire [18, 19]. Both questionnaires consists of (36 resp. 12) multiple choice questions divided over four physical health domains and four mental health domains. The individual scores of all physical health domains (physical function, role limitations due to physical problems, body pain and general health perception) are combined and expressed as physical health score (PHS). In this study we did not include the mental health score. Patients completed HRQoL before and one year after surgery. If a minimum of 50% of the questions was answered in each physical health domain of SF 36-2 and 100% of the questions of SF 12, patients were included in this study.

Based on this PHS, physical recovery was calculated by PHS 1 year after surgery minus baseline PHS. Patients with a score > 0 were included in the physical recovered group (R). Patients with a score ≤ 0 were allocated to the non-recovered group (NR).

### Definition of hospital infections

Patients were categorized into two groups. One group consisted of patients who developed any hospital infection (Infection (I) group). The other group consisted of patients with no hospital infection (Non-Infection (NI) group). Hospital infections were defined as every registered peri- and postoperative infection during hospital stay, according to predefined criteria irrespective of site or severity. These registered infections were deep sternum wound infection (positive cultures and/or surgical drainage and/or antibiotic therapy), pneumonia (positive cultures), urinary tract infection (positive cultures) or arm-/leg wound infection (positive cultures and/or surgical drainage and/or antibiotic therapy [20]. Each of these variables is part of the NHR's mandatory variable set, which has a 90% completeness requirement.

### Statistical analyses

#### Multiple imputation

For the primary analysis, i.e. being the association between hospital infections and non-improvement of PHS adjusted for potential confounders, 2.2% of all values was missing, with the proportion of missing data per variable ranging from 0 to 27.1% (Additional file 1: Table 1). Infection status was missing for n = 67 (0.8%) of patients, and there was no missing data for the outcome variable due to the study design. Since values were assumed to be missing at random, we used multiple imputation to impute 45 datasets using chained equations with imputations drawn using predictive mean matching. For more details, see the Additional file 1. Pooled analysis based on imputed data was used for all analyses in the main paper, unless state otherwise.

#### Descriptive statistics

Normally distributed continuous variables are presented as mean ± standard deviation (SD) and variables with a non-normal distribution as median [interquartile range, IQR]. Categorical variables are described with numbers and percentages. The student's t-test, Mann–Whitney U-test, and the Chi-square test were used to assess differences between the I-group and the NI-group, as appropriate. All analyses were performed using SPSS 24 for Windows^®^ (SPSS INC, Chicago, IL, USA). A *p*-value < 0.05 was considered to be statistically significant. The standardized mean difference (SMD) was calculated as a measure of (im)balance in potential confounders between the NI en I group. Sufficient balance was considered achieved with an SMD < 0.1.

#### Primary study question

In order to obtain an unbiased estimate of the association between hospital infections (NI/I) and physical recovery (NR/R), in the primary analysis, we used binary logistic regression in which the inverse of the propensity score for infection risk was included as a weight variable. Robust sandwich variance estimators were used to deal with the artificially increased sample size by applying weights. In the propensity score model we considered variables marginally associated (*p* < 0.1) with non-recovery as independent variables [21]. Variables that can affect physical recovery, variables both associated with hospital infections and physical non-recovery (confounding factors) and predictive markers described in literature were also included in the model [11].

The final propensity score model with infection as outcome variable included the following independent variables: baseline PHS, unstable angina pectoris, age, gender, extra-cardiac arteriopathy, chronic lung disease, critical preoperative state, DM, NYHA Class III or IV, left ventricular ejection fraction (LVEF), recent myocardial infarction, urgency and surgery on thoracic aorta. More details on the propensity score model can be found in the Additional file 1.

#### Secondary study question

To assess if DM modifies the association between hospital infections and physical recovery, the primary analysis was stratified for DM status. Differential effects of hospital infections on physical recovery for patients with and without DM was formally tested by adding an interaction term (DM*hospital infections) to the main model.

## Results

### Study population

In this study 8577 patients, from all 10 hospitals that report on HRQoL, participated. The median age of all included patients was 69 [IQR = 63–74] years, 75% was male, 52% underwent isolated CABG and 20% underwent urgent surgery. Twenty-six percent of patients were included in NR group. Overall, hospital infections were present in 4.6% of cases.

At baseline, the median pre-operative PHS was slightly, but significantly lower in the infection (I)-group, compared to the NI-group, 53 [37–69] vs 59 [44–74] respectively, *p* < 0.003. Several patient characteristics and individual components of the European System for Cardiac Operative Risk Evaluation (EuroSCORE) II risk model differed significantly between the I- and NI-group (Table 1). This imbalance between the I group and NI group could also be seen in SMD’s which mostly exceed 0.1. These SMD’s were similar to the original un-imputed data (Additional file 1: Table 1).

**Table 1:** Baseline characteristics using the imputed dataset (not weighted)

	All (n = 8577)	NI (n = 8182)	I (n = 395)	*p*-value	SMD (only for variables in the PS model)
*Demographics*					
Baseline PHS	58 [44–74]	58 [44–74]	53 [38–69]	0.003	− 0.237
Body mass index (kg/m^2^)	27 [25–30]	27 [25–30]	28 [25–31]	< 0.001	
*Comorbidities*					
CVA (%)	4.2	4.1	7.1	0.005	
Neurological dysfunction (%)	1.2	1.2	2.8	0.004	
*Cardiac status*					
Unstable AP (%)	1.5	1.5	1.8	0.663	0.021
*EuroSCORE II*					
Age (y)	69 [63–74]	69 [63–74]	71 [66–76]	< 0.001	0.266
Gender male (%)	74.9	75.1	70.9	0.059	0.095
Serum Creat (umol/l)	86 [75–99]	86 [75–99]	90 [77–102]	0.001	
Extra cardiac arteriopathy (%)	8.1	7.7	16.3	0.000	0.264
Poor mobility (%)	3.9	3.9	3.8	0.862	
Previous cardiac surgery (%)	4.4	4.3	5.1	0.451	
Chronic lung disease (%)	10.8	10.4	19.4	0.000	0.256
Active endocarditis (%)	0.6	0.6	0.3	0.518	
Critical preoperative state (%)	0.3	0.3	0.5	0.321	0.003
Diabetes (%)	20.6	20.4	24.7	0.044	0.101
NYHA class III or IV (%)	31.0	30.6	40.7	0.001	0.213
Angina CCS class IV (%)	1.9	1.9	2.4	0.491	
LVEF moderate or poor (%)	23.3	23	28.1	0.022	0.116
Recent MI (%)	14.3	14.2	15.7	0.426	0.041
Pulmonary hypertension (%)	8.0	7.8	10.8	0.035	
Urgency (urgent) (%)	20.1	20	23.5	0.09	0.086
Weight of intervention (%)				0.000	
Isolated CABG	52.0	52.1	50.0		
Single non CABG	25.1	25.5	17.8		
≥ 2 procedures	22.9	22.4	32.3		
Surgery on thoracic aorta (%)	5.1	4.9	9.3		0.172
*Intraoperative characteristics*					
Aortic cross-clamp (min)	60 [42–84]	60 [42–83]	66 [46–99]	< 0.001	
ECC (min)	91 [68–124]	90 [68–123]	102 [71–145]	< 0.001	

Data are presented as median [interquartile range] unless stated otherwise

Angina CCS Class IV, Inability to perform any activity without angina or angina at rest; AP, angina pectoris; CABG, coronary artery bypass grafting; CVA, cerebral vascular accident; ECC, extracorporeal circulation; EuroSCORE, European System for Cardiac Operative Risk Evaluation; I, Infection group; LVEF, left ventricular ejection fraction; MI, myocardial infarction; NI, Non-infection group; NYHA, New York Heart Association; PHS, physical health score; PS, Propensity score; Serum Creat, serum creatinine; SMD, Standardized mean difference

After weighting for the propensity score, SMD’s markedly reduced for all variables and indicated sufficient balance between the I- and NI-group, i.e. all SMD’s being < 0.1. In addition, the median preoperative PHS was also similar between groups.

### Association between hospital infections and recovery

The overall incidence of hospital infections was 4.6%, and this differed between the R and NR groups (4.2 vs 5.5%, respectively). When the types of infection were assessed separately, incidence of lung infection was the most common (2.6 and 3.4% for the R and NR groups, respectively) (Additional file 1: Table 3).

When assessed in the original (un-imputed) dataset without adjustment for confounding (i.e. crude analysis) hospital infections were found to be a risk factor for recovery after cardiac surgery (OR for recovery = 0.76; 95% CI 0.61–0.96; *p* = 0.018). A similar effect estimate was found in the imputed data before weighting. However, after propensity score weighting infection did not remain significantly associated with recovery (OR for recovery = 0.79; 95% CI [0.60–1.03]; *p* = 0.077) (Table 2).

**Table 2:** Associations between hospital infections and recovery in the original dataset and the imputed dataset

	OR recovery	95% CI	*p*-value
Original Data. crude analysis	0.76	0.61–0.96	0.018
Imputed Data. crude analysis	0.75	0.59–0.95	0.017
Imputed Data. weighted* analysis	0.79	0.60–1.03	0.077

*Weights based on inverse of the propensity score for infection risk. The propensity score model included 13 variables: baseline PHS, unstable angina pectoris, age, gender, extra-cardiac arteriopathy, chronic lung disease, critical preoperative state, diabetes, NYHA Class III or IV, LVEF, recent myocardial infarction, urgency and surgery on thoracic aorta

### Associations between hospital infections and recovery in patients with and without diabetes mellitus

Secondly, we assessed the association between hospital infections and recovery in the subgroups of patients with- and without DM. In both groups the association was of similar magnitude as compared to the primary analysis in the whole study population. The interaction analysis further indicated that there was no differential effect of hospital infections on recovery for patients with and without DM (*p* for interaction = 0.845) (Table 3).

**Table 3:** Associations between hospital infections and recovery in patients with and without diabetes mellitus

	OR recovery	95% CI	*p*-value	*p*-value for interaction
No Diabetes	0.78	0.57–1.05	0.101	0.845
Diabetes	0.82	0.50–1.36	0.442	

Analyses were weighted by the inverse of the propensity score for infection risk and performed in the imputed dataset

## Discussion

In this retrospective analysis of a multicenter survey on HRQoL in relation to cardiac surgery, we were unable to convincingly establish a relation between hospital infections and non-recovery one year after the surgical intervention. In addition, there was no differential effect of hospital infections on non-recovery for patients with and without DM.

These findings are seemingly contradictive to our previously reported single-center study, in which hospital infections, DM, female sex, baseline PHS and a coronary re-intervention within one year after surgery all were identified as predictive factors for HRQoL-based physical non-recovery [11]. However, a series of factors need to be taken into account when comparing the two studies. First, the present study defined hospital infections as the primary etiologic variable of interest and its effect on non-recovery, whereas the design of the previous study did not allow for an analysis on etiology, but instead focused on identifying predictive markers [11].To the best of our knowledge, our study is the first in the setting of cardiac surgery to explore hospital infections as an etiologic risk factor for non-recovery, and hence a maximum effort was made to reduce the impact of confounders on this association. Yet, unmeasured confounding cannot be ruled out with the current observational design. Second, the present study included two methods of HRQoL quantification, i.e. the SF36 and the SF12. Although the current literature suggests that both methods are interchangeable [18, 19], such difference must be noted as a potential source of bias. Third, the case mix of both studies was somewhat different. In the current study the percentage of urgent surgery was lower, whereas the percentage of valve surgery is considerably higher in the present study as compared to the previous (25% vs 13%). Nevertheless, in line with our previous work, this study confirms the body of evidence on the high incidence of non-recovery one year after cardiac surgery [11]. Hence, strenuous efforts are needed to further reduce this unwanted outcome after such major surgical intervention.

Based on our findings, we cannot convincingly establish an association between hospital infections and non-recovery one year after surgery. However, with the confidence interval of our main finding (i.e. 0.6–1.03) being close to excluding unity we must interpret our findings with caution. It is well- established that perioperative infections are associated with a substantial clinical and economic burden, including prolonged hospital stay, increase in treatment costs and utilization of medical personnel [22–25]. Moreover, hospital infections are negatively associated with impact on physical and mental health and increased morbidity and mortality [26]. There is no reason to believe that cardiac surgery is an exception to this rule. Recent literature indicates that hospital infections in this setting are associated with increased mortality and costs [27]. However, in general these studies are based on multivariable analyses aimed at risk prediction. Furthermore, we did not observe a differential effect in patients with and without DM, a disease that has been hypothesized to alter the course of infection. Altogether, our findings in the context of literature, do not suggest that hospital infections in the setting of cardiac surgery are irrelevant per se. There may still be subgroups that are increasingly susceptibly to poor prognosis after acquiring a perioperative hospital infection. In addition, there may be differential impact of the different types of hospital infection. Despite having a large cohort of patients, the low incidence of specific types of infection makes it difficult to answer this question convincingly. In a sensitivity analysis, we evaluated specific types of infections (urinary tract infection, arm-leg infection, lung infection, and deep sternal wound infection (DSWI)) as risk factors for non-recovery (Additional file 1: Table 4). This analysis may indeed suggest that acquiring a DSWI may have a stronger effect on non-recovery than lung and urinary tract infections. However, these results should be interpreted with caution due to low numbers. Better selection of patients specifically at risk for hospital infections and subsequent studies, including randomized interventions, seem mandatory to further elucidate the role of hospital infections in the wellbeing of patients.

Our study has several limitations. First, although our data are retrieved from a nationwide survey and are under surveillance of a close-monitoring system we are unaware of the characteristics of non-responders. Second, although the NHR data has a solid quality assurance system with a detailed data manual, containing the definitions and coding guidelines of all the variables collected, defining (hospital) infections remains challenging and leaves room for interpretation. In addition, infection prevention is part of standard patient care, which may vary slightly from hospital to hospital. However, we expect little variation as all protocols are based on national guidelines. Moreover, respiratory- and urinary tract infections often occur a few days after surgery, hence hospital infections could be missed due to transfer back to referring hospitals. Finally, HRQoL questionnaire data are available for 51% of patients for both the pre- and post-operative assessments, and 35% of patients completed the questionnaires at both time points. However, as these questionnaires are not part of the mandatory information of the NHR, and in practice not all patients received the questionnaires, this is at least partially non-selective.

## Conclusions

The present study, a multicenter survey of a general cardiac surgery population, could not convincingly establish hospital infections as a risk factor for non-recovery of HRQoL one year after the surgical intervention. Further research is needed to reduce the substantial percentage of non-recovery.

### Supplementary Information

**Additional file 1.** **Figure 1.** Missing Values. **Supplementary Table 1.** Baseline characteristics of the non-infection (NI) and infection groups (I) in the original dataset. **Supplementary Table 2.** Baseline characteristics of the non-infection (NI) and infection (I) groups in the imputed dataset after propensity score weighting. **Supplementary Table 3.** Baseline and post-operative characteristics of the Recovery and Non-Recovery Group in the original dataset. **Supplementary Table 4.** Associations between specific types of hospital infections and recovery.

## Data Availability

The data underlying this article were provided by The Netherlands Heart Registration by the permission of the participating hospitals. Data are available upon reasonable request to the corresponding author and with permission of the Netherlands Heart Registration.

## Appendix

S. Bramer, Amphia Ziekenhuis, Breda, the Netherlands.

E.J. Daeter, St. Antonius Ziekenhuis, Nieuwegein, the Netherlands.

G.J.F. Hoohenkerk, HagaZiekenhuis, Den Haag, the Netherlands.

A.L.P. Markou, Isala Klinieken Zwolle, the Netherlands.

R.G.H. Speekenbrink, Medisch Spectrum Twente, Enschede, the Netherlands.

P. Segers, Maastricht Universitair Centrum, Maastricht, the Netherlands.

W. Stooker, Onze Lieve Vrouwe Gasthuis, Amsterdam, the Netherlands.

W.W.L. Li, Radboudumc, Nijmegen, the Netherlands.

J.A. Bekkers, Erasmus Medisch Centrum, Rotterdam, the Netherlands.

F. Porta, Medisch Centrum Leeuwarden, the Netherlands.

## Abbreviations

HRQoL: Health related quality of life
DM: Diabetes mellitus
SF 36/-12: Short Form Health Survey 36/-12
NHR: Netherlands Heart Registration
PHS: Physical health score
R-group: Recovery group
NR-group: Non-recovery group
I group: Infection group
NI group: Non-infection group
ESM: Electronic supplemental material
SD: Standard deviation
IQR: Interquartile range
SMD: Standardized mean difference
