# Supplementary material for: Hospital infections and health-related quality of life after cardiac surgery: a multicenter survey
Source: J Cardiothorac Surg. 2024 Feb 10;19:84. doi: 10.1186/s13019-024-02559-4 (PMC10858541; doi:10.1186/s13019-024-02559-4)
Supplement: Supplementary file 1 — Additional file 1. Figure 1. Missing Values. Supplementary Table 1. Baseline characteristics of the non-infection (NI) and infection groups (I) in the original dataset. Supplementary Table 2. Baseline characteristics of the non-infection (NI) and infection (I) groups in the imputed dataset after propensity score weighting. Supplementary Table 3. Baseline and post-operative characteristics of the Recovery and Non-Recovery Group in the original dataset. Supplementary Table 4. Associations between specific types of hospital infections and recovery. [file 13019_2024_2559_MOESM1_ESM.docx]

**Electronic Supplemental Material**

**Hospital infections and Health-Related Quality of Life after cardiac surgery; a multicenter survey**

Hilda G. Rijnhart-de Jong MSc^1,2^*, Jo Haenen MD^1^, Fabiano Porta MD^1^, Marijke Timmermans PhD^4^, E. Christiaan Boerma MD PhD^2,5^ and Kim de Jong PhD^3^ on behalf of the participating centers of the Cardiothoracic Surgery Registration Committee of the Netherlands Heart Registration^†^

**Statistical analyses**

*Missing data and Multiple Imputation:*

Of all variables included in the main analysis, being the exposure “hospital infections”, the outcome “lack of physical recovery” and the variables included in the propensity score model, 2.2% of all values was missing with the proportion of missing data per variable ranging from 0 to 27.1% (ESM Table 1). Of interest, infection status was missing for n = 67 patients (0.8%) and there was no missing data on the study outcome due to the study design. Complete case analysis, i.e. analysis on only those subjects with complete data on each of these variables in the PS model would include 70% of all participants. Hence exclusion of incomplete cases would reduce the dataset and might lead to biased effect estimates.

**Figure 1. Missing Values**


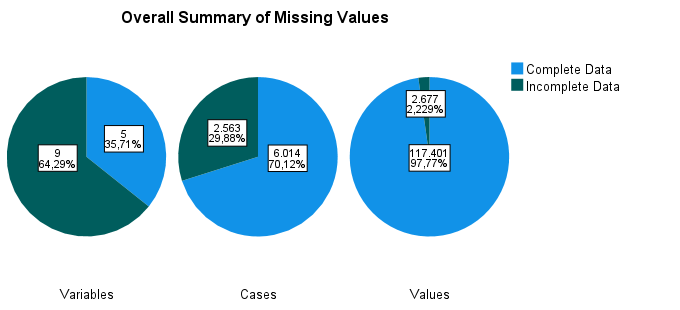


The distribution of missing data appeared to be arbitrary and we assumed missing values to be missing at random. Hence multiple imputation was performed to impute missing values [Collins, 2001 & White, 2011]. We imputed 45 datasets using White’s issues and guidance for practice and chained equations with imputations drawn using predictive mean matching. For each of the 45 dataset we used 50 iterations [White, 2011]. Pooled analysis based on imputed data with weighting for the inverse of the propensity score (see next propensity scores) was used for all analyses except for sensitivity analysis using complete cases.

All variables included in the analysis of the primary study question were imputed and used as predictor for other variables in the imputation model. These were age, gender, DM, left ventricular ejection fraction, extra-cardiac arteriopathy, unstable angina pectoris, NYHA Class III or IV, surgery on thoracic aorta, physical function baseline score, chronical lung disease, critical preoperative state, recent myocardial infarction, urgency, and physical function score 1 year after surgery. Auxiliary variables. i.e. those not included in the main analysis but assumed to be related to missing data in the analysis were also included as predictor in the multiple imputation procedure. Those were body mass index, neurological dysfunction, serum creatinine, poor mobility, previous cardiac surgery, active endocarditis, angina CCS class IV, pulmonary hypertension, weight of intervention, surgical treatment of arrhythmia, aortic cross-clamp time, postoperative cerebral vascular accident, re-sternotomy, readmission ICU and coronary re-intervention. Finally we assumed improvement of the reliability of the dataset over the years because of increasing development in uniformity and performed audits by the NHR. For this reason the variable ‘year’ was also included in the model as predictor.

*Propensity scores:*

Randomized controlled trials are widely accepted as the gold standard for determining causality. In non-randomized studies propensity score methods can be used in an attempt to control for measured confounding and hence to obtain an accurate “unbiased” estimate of the association of interest, i.e. the effect of hospital acquired infections on physical non-recovery.

Subsequently, these propensity scores were used to weigh the “infection effect”, i.e. as 1/PS for subjects with infection and 1/(1-PS) for subjects without infection. We choose this method, i.e. inverse-probability-of-treatment-weighting (IPTW), because we wanted to keep as many patients as possible in the analysis.

The purpose of the propensity scores is to obtain sufficient balance (i.e. marginal or no differences in measured covariates between the group with and without infection). Sufficient balance was defined as standardized differences <0.1.

*References:*

Collins LM, Schafer JL, Kam CM. A comparison of inclusive and restrictive strategies in modern missing data procedures. Psychol Methods. 2001 Dec;6(4):330-51.

White IR, Royston P, Wood AM. Multiple imputation using chained equations: Issues and guidance for practice. Stat Med. 2011 Feb 20;30(4):377-99.

**Supplementary Table 1. Baseline characteristics of the non-infection (NI) and infection groups (I) in the original dataset**

|  | **All***  **(n=8510)** | **NI**  **(n=8118)** | **I**  **(n=392)** | **p-value** | **SMD^#^** | **Missing-ness (%)** |
| --- | --- | --- | --- | --- | --- | --- |
| **Demographics** |  |  |  |  |  |  |
| Baseline PHS | 58 [44-74] | 59 [44-74] | 53 [38-69] | 0.003 | -0.234 | 1.5 |
| Body Mass Index (kg/m^2^) | 27 [25-30] | 27 [25-30] | 28 [25-31] | <0.001 |  | 0.5 |
| **Comorbidities** |  |  |  |  |  |  |
| CVA (%) | 4.1 | 4.1 | 7.0 | 0.004 |  | 2.1 |
| Neurologic dysfunction (%) | 1.2 | 1.2 | 2.8 | 0.009 |  | 1.2 |
| **Cardiac status** |  |  |  |  |  |  |
| Unstable AP (%) | 1.5 | 1.5 | 1.8 | 0.672 | 0.021 | 0.1 |
| **EuroSCORE II** |  |  |  |  |  |  |
| Age (y) | 69 [63-74] | 69 [63-74] | 71 [66-76] | <0.001 | 0.257 | 0.0 |
| Gender Male (%) | 74.9 | 75.1 | 70.9 | 0.062 | 0.094 | 0.0 |
| Serum Creat_s_ (umol/l) | 86 [75-99] | 86 [75-99] | 90 [77-102] | 0.076 |  | 0.1 |
| Extracardiac arteriopathy (%) | 8.2 | 7.8 | 16.3 | <0.001 | 0.265 | 0.1 |
| Poor mobility (%) | 3.8 | 4 | 3.8 | 0.860 |  | 5.0 |
| Previous cardiac surgery | 4.4 | 4.3 | 5.1 | 0.469 |  | 0.0 |
| Chronic lung disease (%) | 10.8 | 10.4 | 19.4 | <0.001 | 0.256 | 0.1 |
| Active endocarditis (%) | 0.6 | 0.6 | 0.3 | 0.518 |  | 0.1 |
| Critical preoperative state (%) | 0.3 | 0.3 | 0.5 | 0.321 | 0.036 | 0.0 |
| Diabetes (%) | 20.6 | 20.5 | 24.7 | 0.043 | 0.102 | 0.4 |
| NYHA Class III or IV (%) | 31 | 30.5 | 40.5 | <0.001 | 0.210 | 27.1 |
| Angina CCS Class IV (%) | 1.8 | 1.9 | 2.4 | 0.492 |  | 4.9 |
| LVEF (moderate or poor (%) | 23 | 23 | 28 | 0.021 | 0.117 | 1.0 |
| Recent MI (%) | 14.3 | 14.3 | 15.6 | 0.462 | 0.037 | 0.1 |
| Pulmonary hypertension (%) | 7.9 | 7.8 | 10.7 | 0.036 |  | 0.1 |
| Urgency (urgent) (%) | 20.2 | 20 | 23.5 | 0.097 | 0.083 | 0.0 |
| Weight of intervention (%) |  |  |  | <0.001 |  | 0.0 |
| Isolated CABG | 52 | 53 | 50 |  |  |  |
| Single Non CABG | 25 | 25 | 18 |  |  |  |
| ≥ 2 Procedures | 23 | 22 | 32 |  |  |  |
| Surgery on thoracic aorta (%) | 5.1 | 4.9 | 9.2 | <0.001 | 0.169 | 0.0 |
| **Intraoperative characteristics** |  |  |  |  |  |  |
| Aortic cross-clamp (min) | 60 [42-84] | 60 [42-83] | 66 [46-99] | <0.001 |  | 8.9 |
| ECC (min) | 91 [68-124] | 90 [68-123] | 102 [71-145] | <0.001 |  | 9.2 |

| * 67 patients (0.8%) excluded due to missing infection data.  # SMD: standardized mean difference, only calculated for variables in the PS model.  Data are presented as median [interquartile range] unless stated otherwise.  Abbreviations: Angina CCS Class IV. Inability to perform any activity without angina or angina at rest; AP. angina pectoris; CABG. coronary artery bypass grafting; CVA. cerebral vascular accident; ECC. extracorporeal circulation; EuroSCORE. European System for Cardiac Operative Risk Evaluation; I. Infection group; LVEF. left ventricular ejection fraction; MI. myocardial infarction; NI. Non-Infection group; NYHA. New York Heart Association; PHS. physical health score; PS. Propensity Score; Serum Creat. serum creatinine. |
| --- |

**Supplementary Table 2. Baseline characteristics of the non-infection (NI) and infection (I) groups in the imputed dataset after propensity score weighting**

|  | **NI**  **(n=8577)** | **I**  **(n=8493)** | **p-value** | **SMD^#^** |
| --- | --- | --- | --- | --- |
| **Demographics** |  |  |  |  |
| Baseline PHS | 58 [44-74] | 59 [43-75] | 0.06 | -0.011 |
| Body Mass Index (kg/m^2^) | 27 [25-30] | 28 [25-31] | <0.001 |  |
| **Comorbidities** |  |  |  |  |
| CVA (%) | 4.1 | 5.6 | <0.001 |  |
| Neurologic dysfunction (%) | 1.2 | 2.6 | <0.001 |  |
| **Cardiac status** |  |  |  |  |
| Unstable AP (%) | 1.5 | 1.4 | 0.409 | -0.008 |
| **EuroSCORE II** |  |  |  |  |
| Age (y) | 69 [63-74] | 69 [64-74] | 0.022 | 0.038 |
| Gender Male (%) | 74.9 | 74.8 | 0.748 | 0.025 |
| Serum Creat_s_ (umol/l) | 86 [75-99] | 87 [76-100] | <0.001 |  |
| Extracardiac arteriopathy (%) | 8.1 | 9.5 | 0.191 | 0.043 |
| Poor mobility (%) | 4.0 | 2.9 | <0.001 |  |
| Previous cardiac surgery (%) | 4.4 | 4.8 | 0.259 |  |
| Chronic lung disease (%) | 10.8 | 10.8 | 0.844 | 0.016 |
| Active endocarditis (%) | 0.6 | 0.6 | 0.671 |  |
| Critical preoperative state (%) | 0.3 | 0.3 | 0.833 | 0.017 |
| Diabetes (%) | 20.6 | 21.1 | 0.518 | 0.010 |
| NYHA Class III or IV (%) | 31.0 | 32.1 | 0.284 | 0.033 |
| Angina CCS Class IV (%) | 1.9 | 1.6 | 0.095 |  |
| LVEF moderate or poor (%) | 23.3 | 23.1 | 0.743 | -0.008 |
| Recent MI (%) | 14.3 | 13.1 | 0.040 | -0.078 |
| Pulmonary hypertension (%) | 7.9 | 10.1 | <0.001 |  |
| Urgency (urgent) (%) | 20.1 | 20.5 | 0.594 | -0.009 |
| Weight of intervention (%) |  |  | <0.001 |  |
| Isolated CABG | 52.0 | 51.3 |  |  |
| Single Non CABG | 25.4 | 20.5 |  |  |
| ≥ 2 Procedures | 22.7 | 28.2 |  |  |
| Surgery on thoracic aorta (%) | 5.1 | 5.4 | 0.531 | 0.022 |
| **Intraoperative characteristics** |  |  |  |  |
| Aortic cross-clamp (min) | 60 [42-84] | 65 [44-94] | <0.001 |  |
| ECC (min) | 90 [67-123] | 99 [67-135] | <0.001 |  |

| # SMD: standardized mean difference, only calculated for variables in the PS model.  Data are presented as median [interquartile range] unless stated otherwise.  Abbreviations: Angina CCS Class IV. Inability to perform any activity without angina or angina at rest; AP. angina pectoris; CABG. coronary artery bypass grafting; CVA. cerebral vascular accident; ECC. extracorporeal circulation; EuroSCORE. European System for Cardiac Operative Risk Evaluation; I. Infection group; LVEF. left ventricular ejection fraction; MI. myocardial infarction; NI. Non-Infection group; NYHA. New York Heart Association; PHS. physical health score; PS. Propensity Score; Serum Creat. serum creatinine. |
| --- |

**Supplementary Table 3. Baseline and post-operative characteristics of the Recovery and Non-Recovery Group in the original dataset**

|  | **All***  **(n=8364)** | **R**  **(n=6198)** | **NR**  **(n=2166)** | **p-value** |
| --- | --- | --- | --- | --- |
| **Demographics** |  |  |  |  |
| Baseline PHS | 58 [44-74] | 55 [42-69] | 69 [54-85] | <0.001 |
| Body Mass Index (kg/m^2^) | 27 [25-30] | 27 [25-30] | 27 [25-30] | 0.969 |
| **Comorbidities** |  |  |  |  |
| CVA (%) | 4.2 | 4.2 | 4.3 | 0.834 |
| Neurologic dysfunction (%) | 1.2 | 1.3 | 1.1 | 0.460 |
| **Cardiac status** |  |  |  |  |
| Unstable AP (%) | 1.5 | 1.7 | 1.0 | 0.021 |
| **EuroSCORE II** |  |  |  |  |
| Age (y) | 69 [63-74] | 69 [63-74] | 70 [63-74] | 0.040 |
| Gender Male (%) | 75 | 75.6 | 74.1 | 0.167 |
| Serum Creat_s_ (umol/l) | 86 [75-99] | 86 [75-99] | 86 [75-100] | 0.601 |
| Extracardiac arteriopathy (%) | 8.2 | 7.7 | 9.5 | 0.009 |
| Poor mobility (%) | 4.0 | 3.9 | 4.2 | 0.522 |
| Previous cardiac surgery | 4.4 | 4.4 | 4.5 | 0.811 |
| Chronic lung disease (%) | 10.7 | 10.3 | 11.8 | 0.046 |
| Active endocarditis (%) | 0.6 | 0.6 | 0.6 | 0.888 |
| Critical preoperative state (%) | 0.3 | 0.2 | 0.5 | 0.039 |
| Diabetes (%) | 20.7 | 20.1 | 22.3 | 0.030 |
| NYHA Class III or IV (%) | 30.8 | 32.1 | 27.2 | <0.001 |
| Angina CCS Class IV (%) | 1.9 | 1.9 | 1.8 | 0.774 |
| LVEF (moderate or poor (%) | 23.2 | 22.6 | 24.8 | 0.037 |
| Recent MI (%) | 14.2 | 13.4 | 16.6 | <0.001 |
| Pulmonary hypertension (%) | 8.0 | 7.9 | 8.2 | 0.736 |
| Urgency (urgent) (%) | 20.1 | 19.4 | 22.0 | 0.011 |
| Weight of intervention (%) |  |  |  | <0.001 |
| Isolated CABG | 51.9 | 52.5 | 50.3 |  |
| Single Non CABG | 25.0 | 24.8 | 25.9 |  |
| ≥ 2 Procedures | 23.0 | 22.7 | 23.9 |  |
| Surgery on thoracic aorta (%) | 5.2 | 4.5 | 7.2 | <0.001 |
| **Intraoperative characteristics** |  |  |  |  |
| Aortic cross-clamp (min) | 60 [42-84] | 60 [42-84] | 60 [41-84] | 0.566 |
| ECC (min) | 91 [68-124] | 91 [68-125] | 91 [67-123] | 0.921 |
| **Postoperative characteristics** |  |  |  |  |
| Infection (%) # | 4.6 | 4.2 | 5.5 | 0.018 |
| Urinary tract | 1.2 | 1.1 | 1.4 | 0.284 |
| Lung | 2.8 | 2.6 | 3.4 | 0.061 |
| Arm- / Leg Wound | 0.3 | 0.3 | 0.3 | 0.992 |
| DSWI | 0.5 | 0.5 | 0.8 | 0.070 |
| Re-sternotomy (%) | 4.3 | 4.0 | 5.3 | 0.010 |
| CVA (%) | 0.6 | 0.4 | 1.3 | <0.001 |
| Readmission ICU (%) | 1.4 | 1.3 | 1.8 | 0.164 |
| Mechanical Ventilation>24 hr (%) | 1.4 | 1.3 | 1.6 | 0.305 |
| Coronary Reintervention (%) | 4.0 | 3.7 | 5.1 | 0.008 |

| * 213 patients (2.5%) excluded due to incomplete SF36-2 data.  # Patients can acquire different types of infections and hence these percentages do not exactly add up to the percentage of patients with any infection (no/yes).  Data are presented as median [interquartile range] unless stated otherwise.  Abbreviations: Angina CCS Class IV. Inability to perform any activity without angina or angina at rest; AP. angina pectoris; CABG. coronary artery bypass grafting; CVA. cerebral vascular accident; DSWI. Deep Sternal Wound Infection; ECC. extracorporeal circulation; EuroSCORE. European System for Cardiac Operative Risk Evaluation; LVEF. left ventricular ejection fraction; MI. myocardial infarction; NR. Non-Recovery group; NYHA. New York Heart Association; PHS. physical health score; R. Recovery group; Serum Creat. serum creatinine. |
| --- |

**Sensitivity analysis: associations between specific types of hospital infections and recovery.**

Associations between specific types of hospital infections and recovery were assessed in the multiple imputed dataset using binary logistic regression in which the inverse of the propensity score for infection risk (composite variable, see methods in main document and ‘*Supplementary data* *Statistical analyses’* in this ESM) was included as a weight variable*.* In contrast to the main analysis, hospital infection (no/yes) was categorized into ‘no’, ‘urinary tract infection’, ‘lung infection’, ‘arm/leg infection’, ‘deep sternal wound infection (DSWI)’, or ‘multiple infections’, the latter in case a patient had more than one type of infection. This variable was modelled as categorical risk factor in the model, with ‘no infection’ being the reference category.

**Supplementary Table 4. Associations between specific types of hospital infections and recovery.**

|  | **% infection *** | |  | |  |
| --- | --- | --- | --- | --- | --- |
| **Type of infection** | **R** | **NR** | **OR recovery** | **95% CI** | **p-value** |
| No infection | 95.8 | 94.6 | reference | reference | reference |
| Urinary tract | 0.99 | 1.16 | 0.77 | 0.46-1.28 | 0.305 |
| Lung | 2.38 | 2.93 | 0.89 | 0.63-1.26 | 0.519 |
| Arm/leg | 0.26 | 0.33 | 0.59 | 0.21-1.63 | 0.308 |
| DSWI | 0.33 | 0.56 | 0.51 | 0.22-1.17 | 0.113 |
| Multiple | 0.23 | 0.37 | 0.66 | 0.25-1.75 | 0.403 |

* % of patients in each infection category, based on the original dataset. These percentages are different as compared to those in ESM table 3, as these categories are mutually exclusive (and in table 3 those are not).

Abbreviations: DSWI. Deep Sternal Wound Infection; R. Recovery group; NR. Non-Recovery group; OR. Odds ratio; 95% CI. 95% confidence interval.
